# The association of ethnicity and migration status with agenda for change pay band in National Health Service healthcare workers: Results from the United Kingdom Research study into Ethnicity and Coronavirus Disease 2019 (COVID-19) Outcomes in Healthcare workers (UK-REACH)

**DOI:** 10.1177/20542704251330157

**Published:** 2025-05-19

**Authors:** Ji Soo Choi, Christopher A. Martin, Lucy Teece, Mayuri Gogoi, Irtiza Qureshi, Daniel Pan, Joshua Nazareth, Rebecca F. Baggaley, Luke Bryant, Padmasayee Papineni, Carol Woodhams, Katherine Woolf, Manish Pareek

**Affiliations:** 1 Department of Respiratory Sciences, 4488 University of Leicester, Leicester, UK; 2 Department of Infection and HIV Medicine, University Hospitals of Leicester NHS Trust, Leicester, UK; 3 National Institute of Health Research (NIHR) Leicester Biomedical Research Centre (BRC), Leicester, UK; 4 Development Centre for Population Health, 4488 University of Leicester, Leicester, UK; 5 Biostatistics Research Group, Department of Population Health Sciences, University of Leicester, Leicester, UK; 6 Nottingham Centre for Public Health and Epidemiology, Nottingham, UK; 7 Li Ka Shing Centre for Health Information and Discovery, Oxford Big Data Institute, University of Oxford, Oxford, UK; 8 WHO Collaborating Centre for Infectious Disease Epidemiology and Control, School of Public Health, Li Ka Shing Faculty of Medicine, The University of Hong Kong, Hong Kong, China; 9 Institute of Health Informatics, 4919 University College London, London, UK; 10 Ealing Hospital, 3749 London North West University Healthcare NHS Trust, Southall, UK; 11 Surrey Business School, 3660 University of Surrey, Surrey, UK; 12 University College London Medical School, London, UK

**Keywords:** ethnicity, migration status, NHS, promotion, pay-scale

## Abstract

**Objectives:**

Ethnic minority and migrant healthcare workers (HCWs) constitute 24% of the UK's National Health Service. Migration status, often overlooked in Human Resources records, is associated with their placement within the Agenda for Change (AfC) pay bands. Therefore, we analysed the association between ethnicity, migration status, and AfC pay bands using data from the UK-REACH cohort study.

**Design:**

Cross-sectional study.

**Setting:**

UK-REACH cohort using baseline data collected via online questionnaires across various healthcare settings.

**Participants:**

Healthcare workers from a broad range of professional roles across the UK, recruited between December 2020 and February 2021.

**Main outcome measures:**

We used multivariable generalised ordered logistic regression models to examine the associations between ethnicity, migration status and AfC pay bands, adjusting for sex, education level, job role, and years qualified.

**Results:**

We found that Overseas-born ethnic minority HCWs were less likely to be in higher AfC pay bands compared to their White UK-born counterparts. Specifically, Asian Overseas-born and Black Overseas-born HCWs reported significantly lower odds of being in higher pay bands compared to White UK-born workers.

**Conclusions:**

Overseas-born HCWs from ethnic minorities resided in lower paid roles than White UK HCWs. Our study is the first to highlight a link between migration status and the AfC pay band and to explore interactions between ethnicity and migration within this context. Our data highlights the need for policymakers to incorporate migration status into NHS-wide electronic records to address career progression and pay inequities.

## Introduction

The National Health Service (NHS) in the United Kingdom (UK), one of the largest employers globally, is composed of a highly diverse workforce with 24% of staff from ethnic minority groups, compared to 18% of the UK population as a whole.^1^

In an effort to standardise pay scales and career pathways for NHS employees, the NHS has implemented a scheme known as Agenda for Change (AfC).^2^ The AfC system objectively categorises NHS employees, excluding doctors, dentists and those in high-level senior management, into grade bands based on their roles and responsibilities.^2^ In line with NHS guidelines, roles at Band 5 and above are typically held by professionally registered individuals (e.g. nurses), while positions in Bands 4 and below are generally administrative or support roles. This classification is supported by NHS job evaluation manuals and workforce research, which show that clinically focused, professionally registered roles are predominantly found at Band 5 and above, although there may be some clinical support roles at lower bands.^3,4^

However, a large-scale study using routinely collected data on AfC has revealed an ethnicity pay gap of 4.6% in favour of White healthcare workers (HCWs) compared to Black/Black British HCWs.^5^

Despite 19.5% of HCWs in the NHS being migrant,^6^ there has been limited investigation into the effects of migration status on the AfC pay band or the interaction between migration status and ethnicity concerning AfC band categorisation. Migrant HCWs may face several challenges that are distinct from non-migrant ethnic HCWs,^7^ such as recognition of international qualifications, limited professional networks, and restricted access to training opportunities, which can potentially impede their career advancement through pay bands. Nevertheless, information on migration status is not routinely collected and thus it is not explored in existing research.

Inferences can be drawn from studies focusing on NHS employees from ethnic minority groups. While 34.3% of the workforce at AfC Band 5 comprised individuals from ethnic minority backgrounds, their representation significantly dropped to 10.3% in bands above AfC Band 5.^8^ This underrepresentation in senior roles means that ethnic minority staff have fewer role models and less influence over administrative and management decisions, such as remuneration or work schedule, which directly affects the quality of the work environment.^9^ The absence of diverse leadership can subsequently impact decisions regarding policy, workplace culture, quality of care and the implementation of anti-discriminatory measures which can lead to policies that do not adequately support ethnic minority HCWs.^10^ This may potentially lead to a higher attrition rate among ethnic minority HCWs within the NHS, which is particularly concerning considering the NHS's heavy reliance on an ethnically diverse workforce amid an ongoing staffing crisis.^11^

We therefore aimed to address gaps in the existing literature by exploring the migration status of the HCWs across different AfC bands. By utilising data from a large nationwide cohort study, our cross-sectional analysis explores the association of ethnicity and migration status with pay band levels, providing initial insights into the pattern of inequalities.

## Methods

### Overview

The United Kingdom Research study into Ethnicity And COVID-19 Outcomes in Healthcare workers (UK-REACH), was established to investigate the impact of the COVID-19 pandemic on UK HCWs from ethnic minority groups. This work represents a secondary data analysis of the baseline questionnaire of the prospective nationwide cohort study administered between December 2020 and March 2021. The cohort study has been described in the published study protocol as well as in previous work using the same dataset.^12,13^ Details of the measures included in the questionnaire can be found in the data dictionary (https://www.uk-reach.org/data-dictionary).

### Study population and recruitment

We recruited HCWs (including ancillary workers in a healthcare setting) aged 16 years or older and/or registered with one of seven UK professional healthcare regulatory bodies (see supplementary information for a list of participating regulators).

We have previously described recruitment into the cohort study.^14^ Briefly, emails with a link to the study website were distributed to HCWs by professional regulators. To take part, eligible HCWs had to visit the website, create a user profile and provide informed consent. The sample was supplemented by recruitment of participants directly through healthcare trusts and advertising on social media/newsletters. We report participation rates as recommended by the Checklist for Reporting Results of Internet E-Surveys (CHERRIES).^15^

### Defining the analysed cohort

This analysis focuses on HCWs on the AfC pay scale and we therefore excluded anyone working in a profession that is not covered by these scales (doctors, dentists, those in senior management positions) and those in other professions who did not report their AfC band. We conducted a complete case analysis, excluding individuals without complete data.

In this study, we hypothesised that the length of time spent in a role would serve as the primary determinant of occupational seniority. While we collected data on the date of professional qualification for participants in professional roles, we did not obtain a comparable measure for individuals in non-professional roles. Therefore, we excluded anyone who was not asked the question relating to the year that they obtained a professional qualification or those with missing data for this variable (see Supplementary Table 2 for details on HCW roles and variable derivations).

Given our focus on including only those with professional qualifications, we anticipated that including individuals in AfC bands below 5 would result in a small sample size as only a few individuals with professional qualifications are placed in bands below 5. Therefore, to avoid creating a heterogeneous category by collapsing this small subgroup into the lowest band, we opted to exclude these individuals from the analysis.

Additionally, our recruitment strategy predominantly involved professional regulators, resulting in a higher proportion of professional staff compared to non-professional staff. This distribution provided greater statistical power for analysing AfC banding within the professional groups.

### Outcome measure

Our outcome measure was AfC pay band at the time of the baseline questionnaire. We derived a four-level variable (1. Band 5; 2. Band 6; 3. Band 7; 4. Band 8 and above) collapsing those in bands over 8 into the highest category due to low numbers in these groups.

### Exposure

We were primarily interested in exploring the association between ethnicity and migration status with the outcome. For ethnicity, we used the five aggregated categories (White, Asian, Black, Mixed, Other) used by the Office for National Statistics (ONS).^16^ For migration status we used a binary variable indicating whether the participant reported being born in the UK or being born overseas. To investigate the interaction between ethnicity and migration status we combined these measures into a 10-level variable (as described in previous analyses), splitting each of the five ethnic groups into two according to migration status. Hereafter, we refer to these groups as the ethnic group followed by an indication of whether they were born in the UK or overseas (e.g. Asian UK, Black overseas).

In a sensitivity analysis, we explored the impact of being trained abroad. The aim was to determine whether those born abroad and trained in the UK experience similar impacts as those both born and trained abroad, or whether there are graded differences between the two groups. Those born in the UK but trained abroad were small in number and excluded from the sensitivity analysis.

### Covariates

We selected covariates that might confound the association between our exposures and outcome *a priori*. These included the number of years an HCW reported having held their professional qualification, self-reported sex, educational level (undergraduate or below, masters or doctorate) and occupational group (nursing, allied health professionals, pharmacy workers, healthcare scientists, ambulance workers and those in optical roles). Age was predicted to be highly collinear with years qualified and was therefore not included in models.

### Statistical analysis

We summarised non-normally distributed continuous variables as median and interquartile range (IQR) and categorical variables as frequency and percentage.

We examined contingency tables of our exposure and other covariates cross-tabulated with our outcome measure and compared distributions using Kruskal-Wallis tests for continuous variables and chi-squared tests for categorical variables (Supplementary Figure 5).

As our outcome is an ordered categorical variable, and we anticipated that certain variables (e.g. job) would violate the proportional odds assumption if ordered logistic regression was employed, for multivariable analysis we used generalised ordered logistic regression (using the Stata command: *gologit2* with the autofit option).^17^ This is equivalent to a series of binary logistic regressions where categories of the dependent variable are dichotomised at each cut-off and the coefficients for each variable that would violate the proportional odds assumption are allowed to vary across the categories of the outcome variable. Each level was compared to the next higher level and defined as a cut point. For instance, cut point 1 compared Band 5 to Band 6 and above, while cut point 2 compared Band 6 and below to Band 7.

We present the results of this analysis as adjusted odds ratios, 95% confidence intervals (CIs) and *p* values for each variable at each level of the outcome. We also calculated average marginal effects (and their 95% CIs) for each ethnic/migrant group setting years qualified at 5-year intervals between 0 and 40 years) we then plotted the probability of AfC band membership over years qualified by ethnic/migrant group. Odds Ratios for each cut point were fixed for ethnicity, migration status, interaction and sex, but varied for years qualified, job role and educational level.

We first examined the effects of ethnicity and migration status independently and then the interaction (using the 10-level ethnicity/migration status variable described above). Those from ‘Other’ ethnic groups were excluded from the latter analysis due to low numbers in this group.

All analyses were performed in Stata version 17 for macOS (StataCorp, College Station, Texas, USA).

## Results

### Formation of the analysed sample

After excluding participants with missing data on ethnicity and migration status, a total of 5771 participants were included in the analysis (Supplementary Figure 1). Subsequently, 2.66% of the remaining participants had missing data for covariates and were therefore excluded from the analysis.

### Description of the analysed cohort

Table 1 provides a summary of the demographic details of this cohort. The majority of the participants were female (84.4%), UK-born (81.8%), and of White ethnic origin (82.3%). Among the remaining participants, 10.2% were Asian, 3.2% were Black, and 3.3% were of mixed ethnic origins. In terms of professional roles, 36.3% were nurses, nursing assistants, or midwives, 45.9% were allied health professionals (AHPs), and 8.3% were healthcare scientists.

**Table 1.** Description of the analysed cohort.

Variable	Total, *n* (%)
Sex	
Female	4870 (84.4)
Male	901 (15.6)
Ethnicity	
White UK	4258 (73.8)
White Overseas	493 (8.5)
Asian UK	242 (4.2)
Asian Overseas	344 (6.0)
Black UK	67 (1.2)
Black Overseas	119 (2.1)
Mixed UK	147 (2.5)
Mixed Overseas	45 (0.8)
AfC band	
Band 5	1166 (20.2)
Band 6	2154 (37.3)
Band 7	1575 (27.3)
Band 8 or above	876 (15.2)
Migration status	
UK born	4722 (81.8)
Overseas born	1049 (18.2)
Training location	
Born and trained in the UK	4705 (81.5)
Born Overseas, trained in the UK	527 (9.1)
Born and trained Overseas	522 (9.0)
Occupation	
Nurse, NA, midwives	2095 (36.3)
AHPs	2648 (45.9)
Pharmacy	128 (2.2)
Healthcare scientist	480 (8.3)
Ambulance	382 (6.6)
Optical	38 (0.7)
Educational level	
Undergrad or lower	3906 (67.7)
Masters	1582 (27.4)
Doctorate	283 (4.9)
Years qualified	Median (IQR)
White	17 (8, 29)
Asian	15 (6, 24)
Black	15 (7, 25)
Mixed	12 (5, 20)
Other	15 (6, 28)

Figures are described in percentages and absolute numbers (*n*) unless otherwise stated. For a detailed description of the cohort by ethnicity, see Supplementary Table 3. NA = nursing associate, AHP = advanced healthcare practitioner.

### Ethnicity

**Figure 1.**
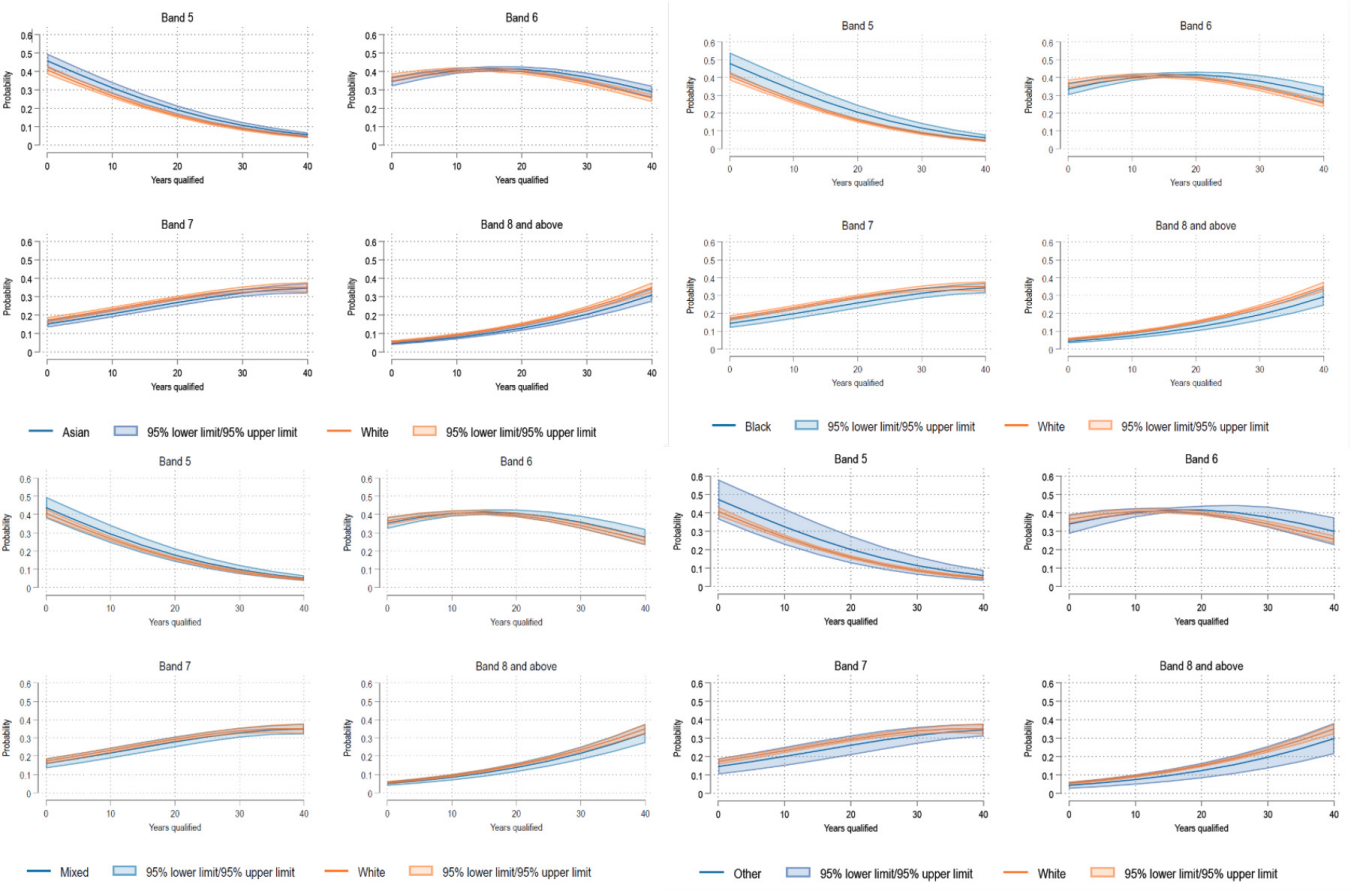
Average marginal effects over the years qualified for each ethnic group: Asian (top left), Black (top right), mixed (bottom left), and White (bottom right).

Adjusted odds ratios (aOR) for each ethnic group did not vary across the cut points of the four-level AfC band outcome variable. After adjustment for sex, education level and occupation, Asian and Black HCWs had reduced odds of being in a high AfC band compared to White HCWs (Asian: aOR 0.79, 95% CI 0.67- 0.93, *p* < .005; Black: aOR 0.71, 95% CI 0.54–0.94, *p* < .017). Figure 1 shows a plot of average marginal effects over years qualified for each ethnic group. After being qualified for 10 years, Black HCWs had a predicted probability of 0.33 (95%CI 0.28–0.38) for being in Band 5, compared to 0.27 (95%CI 0.25–0.28) for White HCWs. After being qualified for 30 years, Black HCWs had a predicted probability of 0.19 (95%CI 0.16–0.23) for being in Band 8 and above, compared to 0.23 (95%CI 0.22–0.25) for White HCWs.

### Migration status

**Figure 2.**
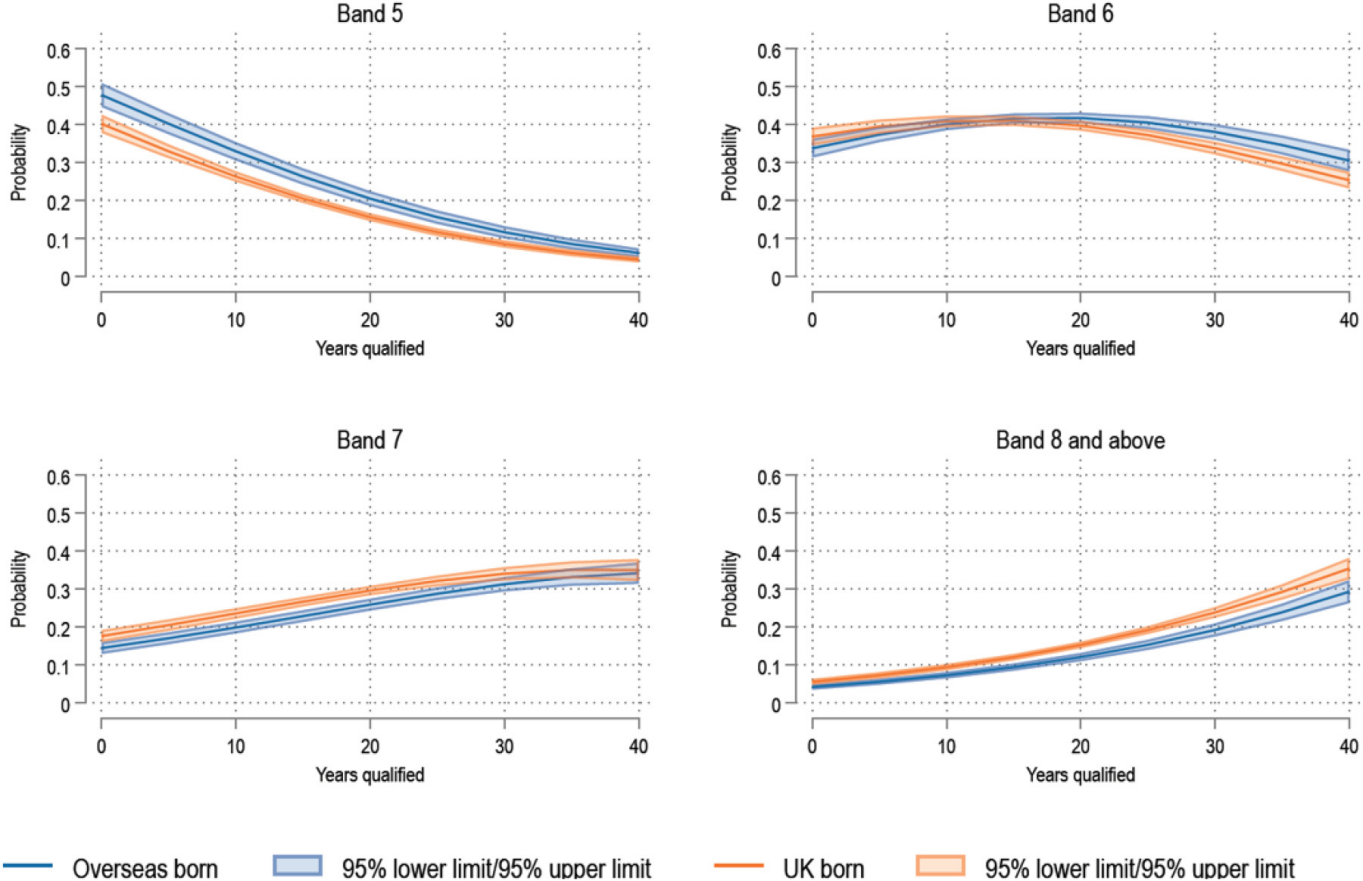
Average marginal effects over years qualified for Overseas and UK-born HCWs.

As with ethnicity, aORs for migration status did not vary across cut points of the outcome. HCWs born overseas had lower odds of reporting being in higher bands compared to their UK-born counterparts (aOR 0.69, 95% CI 0.61–0.79, *p* < .001). This trend of reduced odds was consistent across all cut points of the bands analysed. Figure 2 shows a plot of average marginal effects over years qualified for overseas-born and UK-born. After being qualified for 10 years, the predicted probability of being in Band 5 was 0.33 (95%CI 0.30–0.35) for overseas-born HCWs, compared to 0.26 (95%CI 0.25–0.28) for UK-born HCWs. After being qualified for 30 years, the predicted probability of being in Band 8 and above was 0.19 (95%CI 0.17–0.21) for Overseas born HCWs, compared to 0.24 (95%CI 0.22–0.25) for UK-born HCWs.

### Interaction of ethnicity and migration status

**Figure 3.**
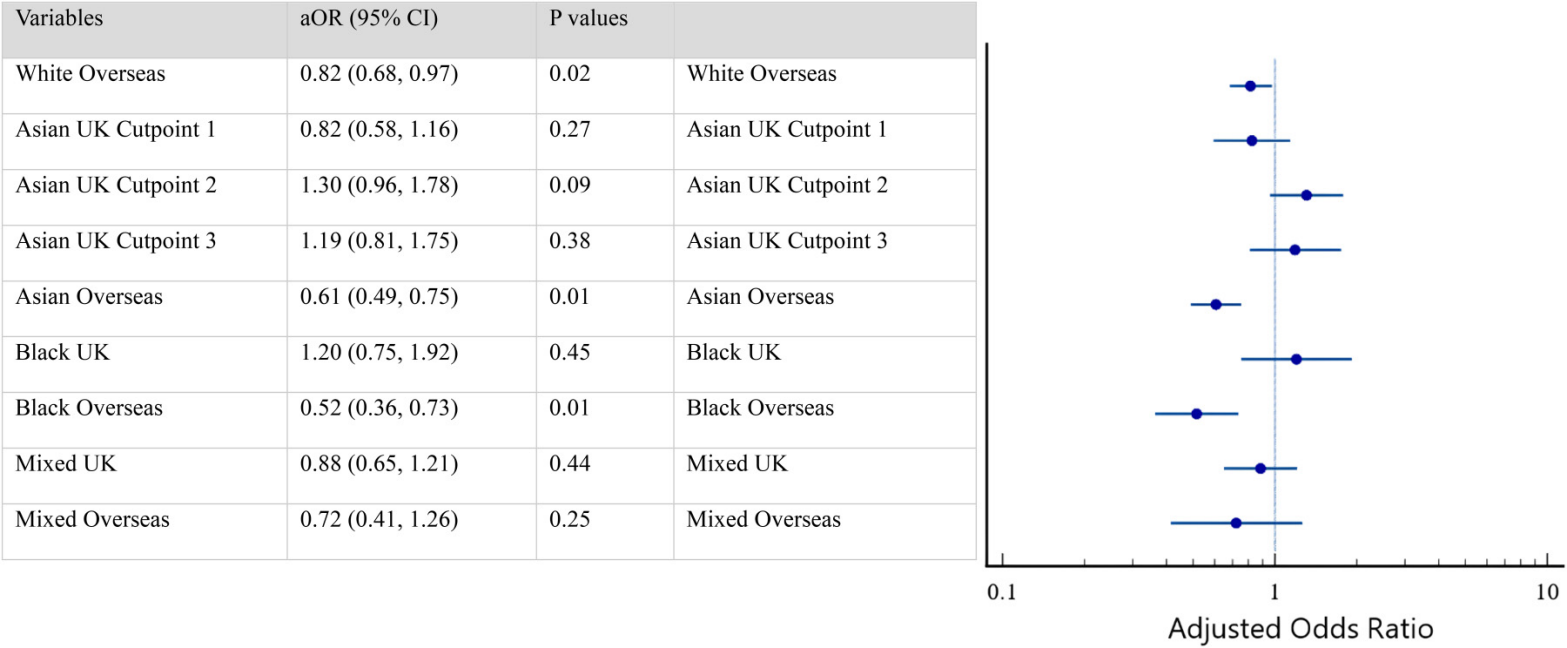
Forest plot.

Figure 3 presents a summary of a generalised ordered logistic regression analysis that explores the association between ethnicity and AfC bands. The analysis shows consistent adjusted odds ratios across cut points for White overseas-born, Asian overseas-born, Black UK-born, and Black overseas-born individuals. Odds ratios for Asian UK-born were allowed to vary across cut points; however, there were no significant differences between this group and the White UK-born group at any level of the outcome. In contrast, Asian overseas-born HCWs were less likely to be in higher bands compared to White UK-born HCWs (aOR 0.61, 95% CI 0.49–0.75, *p *< .001). There were no significant differences between Black UK-born and White UK-born groups (aOR 1.20, 95% CI 0.75–1.92, *p *< .449), while Black overseas-born individuals had approximately half the odds of being in higher bands compared to White UK-born individuals (aOR 0.52, 95% CI 0.36–0.73, *p *< .001). White overseas-born HCWs were less likely to be in higher bands compared to their White UK-born counterparts (aOR 0.82, 95% CI 0.68–0.97, *p *< .024).

Forest plot showing adjusted odds ratios (aORs), 95% confidence intervals (95% CI) and *p*-value across different cut points for ethnicity, migration status and years qualified. aORs were consistent across cut points for each ethnicity and migration status, except for Asian UK, where they varied across three different cut points as above. For a detailed description of the cohort including job roles and qualification, see Supplementary Table 4.

**Figure 4.**
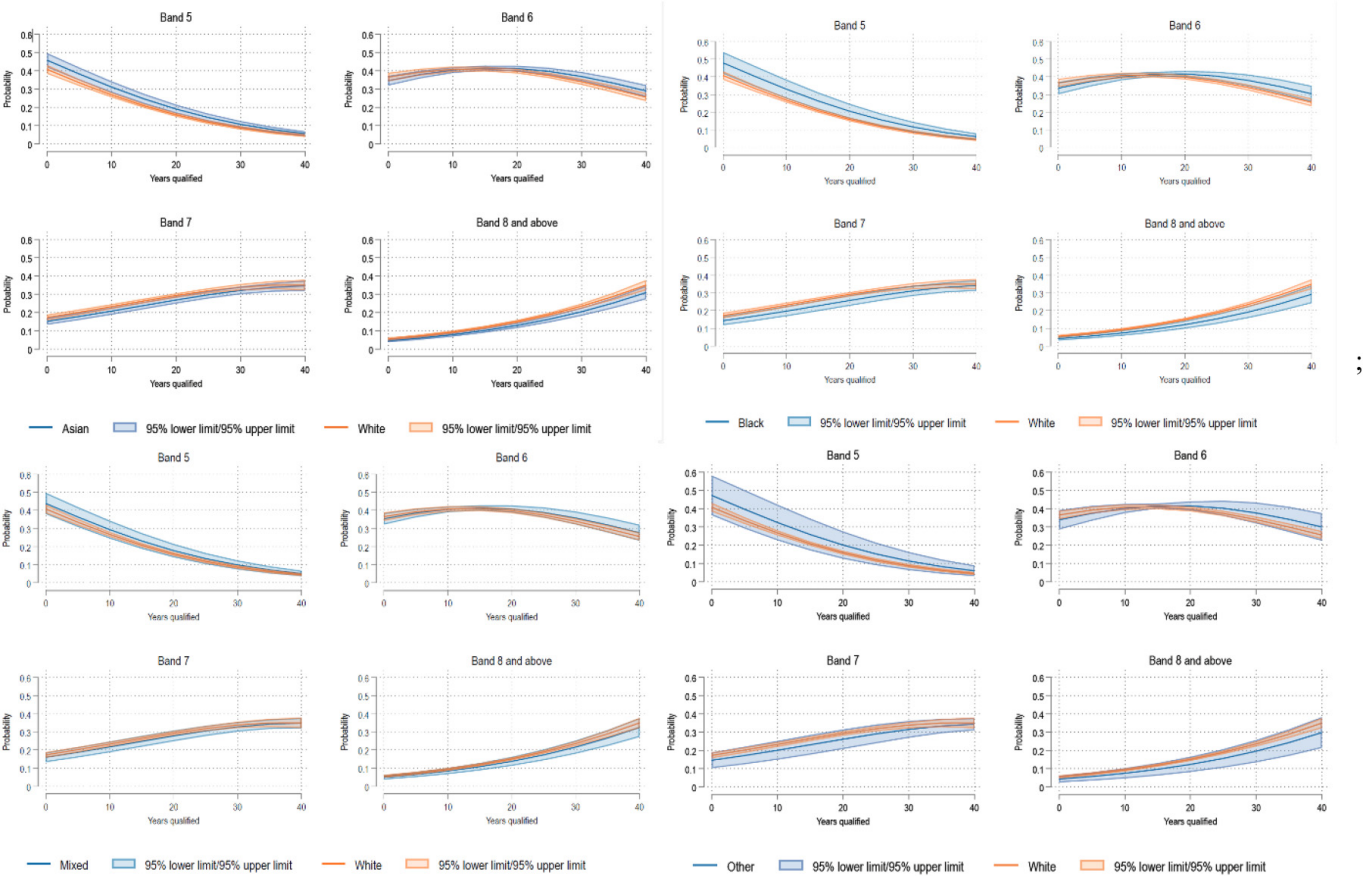
Average marginal effects over years qualified for each ethnic group and migration status: Asian (top left), Black (top right), mixed (bottom left), and other (bottom right).

Figure 4 shows a plot of average marginal effects over years qualified for each ethnic group. After 10 years of qualification, the probability of being in Band 5 was 0.39 (95%CI 0.32–0.46) for Black overseas-born HCWs and 0.23 (0.16–0.31) for Black UK-born HCWs. Asian UK-born HCWs had an estimated probability of 0.30 (95%CI 0.23–0.36) for being in Band 5 after 10 years, this compares to 0.35 (95%CI 0.31–0.39) for Asian overseas-born individuals. After 30 years, the probability of being in Band 8 and above was higher for Asian UK-born HCWs (0.26, 95%CI 0.20–0.32) compared to Asian overseas-born HCWs (0.18, 95%CI 0.15–0.20) and for Black UK-born (0.26, 95%CI 0.19–0.33) compared to Black overseas-born (0.16, 95%CI 0.12–0.19) HCWs.

### Country of training

Adjusted odds ratio (aOR) for individuals born overseas and trained in the UK was similar to those born and trained in the UK, remaining consistent across all three cut points (aOR 0.75, 95% CI 0.63–0.89, *p *< .001). For those born and trained abroad, their likelihood of being in Bands 5 or below was similar to those born overseas and trained in the UK (aOR 0.75, 95% CI 0.63–0.89, *p *< .001). However, their likelihood of being in Band 6 or above was halved (aOR 0.54, 95% CI 0.44–0.68, *p *< .001), although this likelihood improved at Band 7 or above (aOR 0.75, 95% CI 0.63–0.89, *p *< .001).

## Discussion

In this study of a national cohort of healthcare workers, we analysed the association of ethnicity and migration status with position in the NHS Agenda for Change (AfC) framework. Our findings indicate that both ethnicity and migration status were associated with a greater likelihood of occupying lower AfC band placements. Crucially, when studying the interaction between ethnicity and migration status, differences were greater between White UK-born and Asian or Black overseas-born HCWs, with the latter groups being more likely to report occupying lower bands. Similarly, those who were born and trained in the UK were most likely to report occupying higher bands compared to those who were born and trained in Overseas.

Our results are consistent with previous literature which indicates that ethnic minority HCWs are disproportionately represented in lower pay grades and face barriers to progress beyond entry-level positions.^8,18^ Woodhead *et al.* report that systemic biases and discriminatory practices are prevalent, impacting not only direct career advancement but also indirectly through fatigue and resignation among minority staff.^10,19^ This discourages attempts to develop, reinforcing the state of static progress.^7^ Wingfield et al. further argues that resistance to ethnic minority staff is further perpetuated by the presence of racialised hierarchies in the workplace.^20^ The reasons behind this disproportionate representation include societal racism which leads to institutional practices that maintain racial hierarchies and create disparate outcomes for minority HCWs.^20^

At the organisational level, ethnic minorities are underrepresented at senior managers and trustees on NHS boards.^21^ Resulting policies and regulations may appear race-neutral but can have a disproportionately negative impact on ethnic minority HCWs, and in particular, Black workers. For instance, restrictions on certain hairstyles for workers (e.g. dreadlocks, braids) are supposedly creating policies that apply to all employees, but in practice have a disproportionate impact on Black culture.^22^ Such chronic challenges lead to a cumulative detrimental effect on the well-being and career trajectories of these staff members.^23,24^

Furthermore, ethnic minority HCWs were more than 1.2-2.0 times as likely as White staff to be subjected to the formal disciplinary process in 70% of NHS trusts, with this relative likelihood exceeding 3.0 in 20 NHS Trusts across the UK.^8^ One of the contributors to this is possibly due to a lack of training and mentoring of ethnic minority HCWs to understand expected behaviour, leading to subjective behaviours that do not conform to norms. This issue is compounded by the lack of ethnic minority staff in senior positions to challenge stereotypes which often leads to unfair decision-making at various stages of the disciplinary process.^25^ Likewise, women from White Gypsy or Irish Traveller backgrounds faced the most severe harassment, and abuse from patients and the public,^8^ suggesting that the discrimination may stem from factors beyond ethnicity.

Uncoupling migration status from ethnic status, in Band 5 and above, these levels are associated with higher behavioural competencies, as defined by the NHS Knowledge and Skills Framework (KSF)^26^ underpinning AfC. This job evaluation framework forms the foundation for selection and promotion processes across the NHS. However, at these higher levels, there is a heightened risk of divergent cultural interpretations of leadership skills and competencies, which may lead to inconsistent evaluations. Despite AfC being a robust and comprehensive system that has undergone scrutiny to address potential inequities, it remains influenced by culturally rooted evaluations of interpersonal and leadership qualities. This raises questions regarding structural and normative barriers to progression, particularly for migrant workers.

In addition, the challenges faced by those born abroad but trained in the UK differ markedly from those encountered by individuals who were internationally recruited and migrated to the UK for economic reasons.^27,28^ These barriers, such as visa processes and unfamiliarity with NHS-specific practices, may contribute to the fact that migration status can present more hurdles to advancement compared to UK-born ethnic minorities, especially in the higher AfC bands. This suggests that further investigation is needed into how these cultural and structural factors intersect to produce unequal progression outcomes, with a focus on how migrant and ethnic minority staff are perceived within leadership frameworks.

The implications of our findings are complex. Our findings suggest that migration status may be more strongly associated with AfC pay band disparities than is ethnicity, yet it remains a substantially overlooked aspect. Efforts to address pay inequities will continue to be hindered until data on intersectional characteristics are collected and made available to analysts. The lack of data on this issue may stem from current Human Resources systems, which do not routinely collect information on migration status, meaning it often cannot be included in assessments of the intersecting factors contributing to systemic inequalities.^29^

Furthermore, a more granular analysis of the origin and country of training of individuals may reveal insights into its impact on their placement within the AfC bands. Although our current dataset lacks the numbers to conduct such an analysis, a broader NHS-wide study could provide valuable insights.

Our study is not without its limitations. The nature of the electronic questionnaire may introduce selection bias, as individuals who complete research surveys might be expected to be more senior workers with greater administrative responsibilities. Additionally, the way the UK-REACH study was marketed as ethnicity-focused could further skew participation rates by ethnicity. Our data compared to the publicly available NHS data from the WRES report 2022^8^ shows a discrepancy in the proportion of ethnic minority staff in clinical AfC roles. The WRES report indicates that 38.9% of ethnic minority staff are at Band 5 and 15.6% at Band 8. However, our UK-REACH Cohort shows 20.9% at Band 5 and 20.0% at Band 8. This suggests that our data may not fully reflect the underrepresentation of ethnic minority staff in higher bands as observed in NHS data, potentially giving an overestimate of the progress ethnic minorities are making. Together, these factors could introduce selection biases that might alter the results.

Moreover, our study excluded individuals in AfC bands below 5 from the analysis. While the focus on professionally qualified HCWs allowed a more homogeneous analysis of occupational seniority within professional roles, the absence of data from non-professional HCWs restricts the generalisability of our findings to the broader NHS workforce. Non-professional roles, which include support and administrative positions, play a crucial part in the functioning of healthcare systems. By excluding this group, we miss the opportunity to explore how occupational seniority and AfC banding may differ between professional and non-professional staff, limiting our understanding of the full spectrum of NHS roles. Including individuals from bands below 5 would have provided a more comprehensive view of workforce dynamics, potentially revealing insights into the contributions and career progression of non-professional staff within the NHS.

Therefore, future research should prioritise including migration status as a standard data point collected alongside other demographic information in non-selective, representative NHS cohorts. Such insights into the intersections of identity categories can shed light on their impact on individuals’ experiences and career opportunities.^29^ This enhanced data granularity not only addresses the current workforce crisis by capturing a wider spectrum of identity factors but also encourages researchers and policymakers to develop interventions for more equitable outcomes.

Additionally, qualitative methodologies could provide deeper insight into the personal experiences of migrant ethnic minority workers in the NHS. Conducting interviews or focus groups can offer a richer understanding of the barriers they face and reveal the different perceptions and experiences that quantitative methods might overlook. These narratives can communicate the day-to-day challenges these workers face and the personal impact of systemic barriers on their professional and personal lives.^30^

## Conclusion

Our findings showed that there may be an association between ethnicity, migration status and lower placement within AfC bands, which highlights the need for NHS-wide data to investigate the impact of migration status in a larger, non-selected cohort. This is particularly important given the demographic composition of the NHS and the lack of capacity due to the ongoing workforce crisis. Future studies must also seek to understand the impact of ethnicity and migration status in those in non-professional roles and in AfC bands below 5. By gaining these insights, healthcare organisations can enhance equity and retain their full potential of their diverse workforce, ultimately sustaining workforce capacity, improving service delivery and patient care.

## Supplemental Material

sj-pdf-1-shr-10.1177_20542704251330157 - Supplemental material for The association of ethnicity and migration status with agenda for change pay band in National Health Service healthcare workers: Results from the United Kingdom Research study into Ethnicity and Coronavirus Disease 2019 (COVID-19) Outcomes in Healthcare workers (UK-REACH) Supplemental material, sj-pdf-1-shr-10.1177_20542704251330157 for The association of ethnicity and migration status with agenda for change pay band in National Health Service healthcare workers: Results from the United Kingdom Research study into Ethnicity and Coronavirus Disease 2019 (COVID-19) Outcomes in Healthcare workers (UK-REACH) by Ji Soo Choi, Christopher A. Martin, Lucy Teece, Mayuri Gogoi, Irtiza Qureshi, Daniel Pan, Joshua Nazareth, Rebecca F. Baggaley, Luke Bryant, Padmasayee Papineni, Carol Woodhams, Katherine Woolf and Manish Pareek in JRSM Open

sj-doc-2-shr-10.1177_20542704251330157 - Supplemental material for The association of ethnicity and migration status with agenda for change pay band in National Health Service healthcare workers: Results from the United Kingdom Research study into Ethnicity and Coronavirus Disease 2019 (COVID-19) Outcomes in Healthcare workers (UK-REACH) Supplemental material, sj-doc-2-shr-10.1177_20542704251330157 for The association of ethnicity and migration status with agenda for change pay band in National Health Service healthcare workers: Results from the United Kingdom Research study into Ethnicity and Coronavirus Disease 2019 (COVID-19) Outcomes in Healthcare workers (UK-REACH) by Ji Soo Choi, Christopher A. Martin, Lucy Teece, Mayuri Gogoi, Irtiza Qureshi, Daniel Pan, Joshua Nazareth, Rebecca F. Baggaley, Luke Bryant, Padmasayee Papineni, Carol Woodhams, Katherine Woolf and Manish Pareek in JRSM Open
