# Corrigendum to “The association of ethnicity and migration status with agenda for change pay band in National Health Service healthcare workers: Results from the United Kingdom Research study into Ethnicity and Coronavirus Disease 2019 (COVID-19) Outcomes in Healthcare workers (UK-REACH)”

**DOI:** 10.1177/20542704251363054

**Published:** 2025-07-23

Choi JS, Martin CA, Teece L, et al. The association of ethnicity and migration status with agenda for change pay band in National Health Service healthcare workers: Results from the United Kingdom Research study into Ethnicity and Coronavirus Disease 2019 (COVID-19) Outcomes in Healthcare workers (UK-REACH). *JRSM Open*. 2025;16(5). doi:10.1177/20542704251330157

The author reported that Figure 4 was incorrect in the published version of the article. It has now been corrected and the article has been updated with the new figure to reflect accuracy of the research findings.

