# Supplementary material for: The association of ethnicity and migration status with agenda for change pay band in National Health Service healthcare workers: Results from the United Kingdom Research study into Ethnicity and Coronavirus Disease 2019 (COVID-19) Outcomes in Healthcare workers (UK-REACH)
Source: JRSM Open. 2025 May 19;16(5):20542704251330157. doi: 10.1177/20542704251330157 (PMC12089724; doi:10.1177/20542704251330157)
Supplement: sj-pdf-1-shr-10.1177_20542704251330157 - Supplemental material for The association of ethnicity and migration status with agenda for change pay band in National Health Service healthcare workers: Results from the United Kingdom Research study into Ethnicity and Coronavirus Disease 2019 (COVID-19) Ou [file sj-pdf-1-shr-10.1177_20542704251330157.pdf]

Table of contents

Supplementary Figure 1. Formation of the analysed sample 2

Supplementary Table 2. Derivation of variables from questionnaire data..................... 3

Supplementary Table 3. Contingency table of exposure and other covariates.................5

Supplementary Table 4. Detailed description of variables....................................... 6

Supplementary Figure 1. Formation of the analysed sample

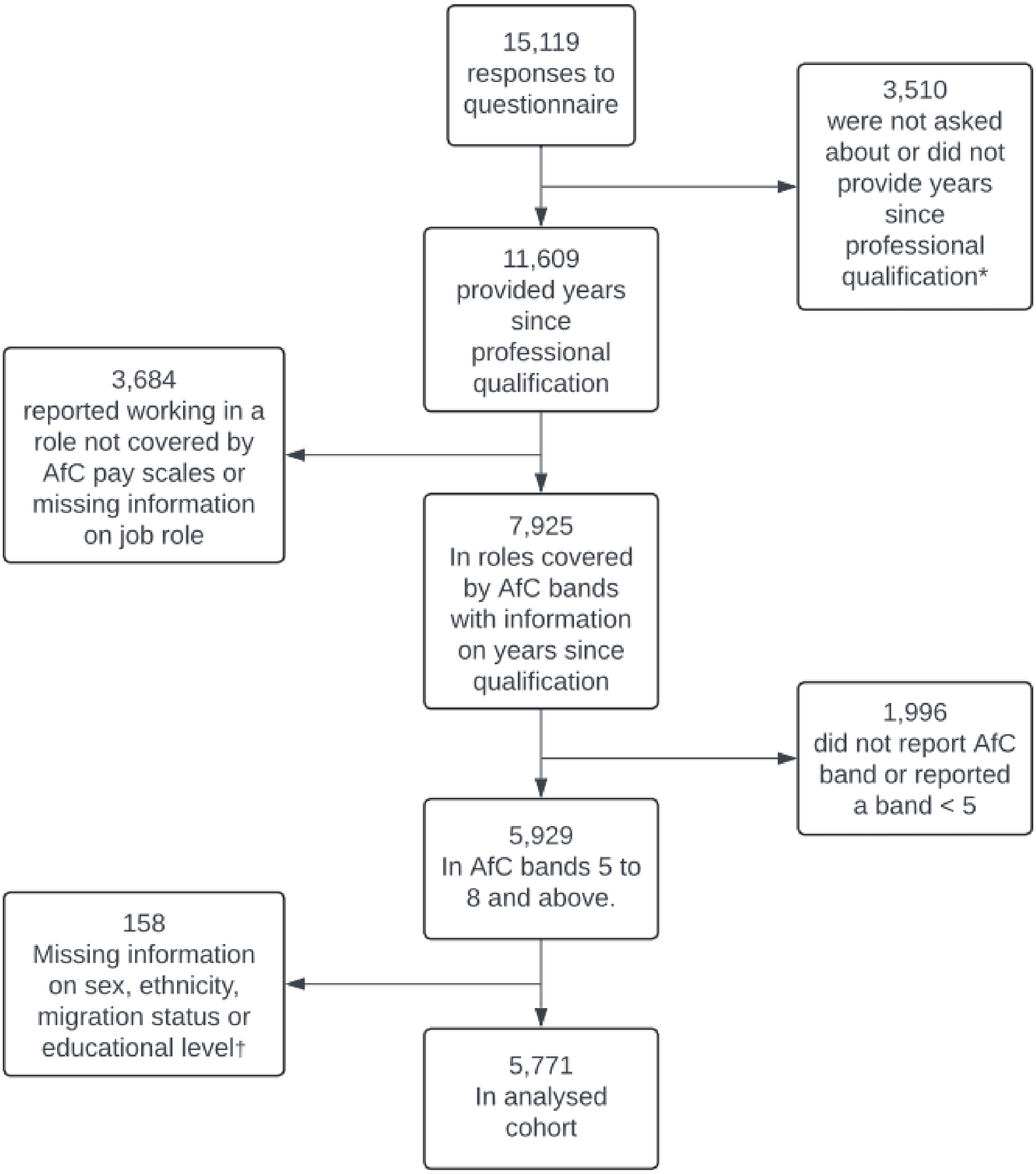

Supplementary Figure 1.
Illustrates the process of selecting the analysed sample from participants who completed the UKREACH questionnaire.

Supplementary Table 2. Derivation of variables from questionnaire data

Variable	Description
**Sex**	Binary variable. Participants were asked their sex assigned at birth.
**Ethnicity**	Categorical variable. Participants were asked to select their ethnicity from a list of the 18 Office for National Statistics categories (2011):<br><br>Asian/Asian British – Indian<br><br>Asian/Asian British – Pakistani<br><br>Asian/Asian British – Bangladeshi<br><br>Asian/Asian British – Chinese<br><br>Asian/Asian British - Any other Asian background<br><br>Black/African/Caribbean/Black British - African<br><br>Black/African/Caribbean/Black British – Caribbean<br><br>Black/African/Caribbean/Black British - Any other Black/African/Caribbean background Mixed/Multiple ethnic groups - White and Black Caribbean<br><br>Mixed/Multiple ethnic groups - White and Black African<br><br>Mixed/Multiple ethnic groups - White and Asian<br><br>Mixed/Multiple ethnic groups - Any other Mixed/multiple ethnic background<br><br>White - English/Welsh/Scottish/Northern Irish/British<br><br>White – Irish<br><br>White - Gypsy or Irish Traveller<br><br>White - Any other white background<br><br>Other ethnic group – Arab<br><br>Other ethnic group - Any other ethnic background<br><br>These were categorised into the 5 broader Office for National Statistics ethnicity categories (Asian, Black, Mixed, White, Other).
**Migration status**	Binary variable. Participants were asked whether they were born in the UK.
**Ethnicity and migration status**	Categorical variable. Each of the 5 broad ethnic groups were further split into 2 groups based on migration status (born in the UK vs born abroad) to create a 10-level variable.
**Migration status and place of training**	Categorical variable. We first derived a binary variable for place of training (UK vs abroad) from responses to the question "In which country did you gain your primary professional qualification?". Participants could select from any country in the world. We then combined this with the migration status variable to derive a three level variable: Born and trained in the UK, Born abroad and trained in the UK, Born and trained abroad. Those who were born in the UK and trained abroad were small in number and excluded from the sensitivity analyses using this variable.
**Occupation**	Categorical variable. Participants were asked to select their main job/role. Categorised as below:<br><br>**Nursing -** Advanced Nurse Practitioner, Midwife, Nurse, Nursing Associate, Other nursing and midwifery role<br><br>**Allied Health Professional -** Arts therapist, Chiropodist/Podiatrist, Dietician, Occupational therapist, Operating department practitioner, Orthoptist, Physiotherapist, Practitioner psychologist, Prosthetist / Orthotist, Radiographer, Speech and language therapist, Other Allied Health Professional role<br><br>**Pharmacy –** Pharmacist, Pharmacy technician, Other pharmacy role

	**Healthcare scientist –** Biomedical scientist, Clinical scientist<br><br>**Ambulance - Paramedic**<br><br>**Optical** - Dispensing optician, Optometrist, Other Optical role<br><br>Note: this only shows the job roles reported by participants in the final analysis. For a full list of job roles included in the UK-REACH questionnaire please see the data dictionary (https://uk-reach.org/main/data-dictionary/)
**Agenda for change band**	Categorical variable. Participants who reported working in roles covered by the agenda for change bands were asked to report their current band. Participants reporting bands lower than 5 were excluded (as very few who hold a professional qualification were working in bands lower than band 5). Those working in any of the band 8 strata (a – d) or band 9 were collapsed into a category of "band 8 and above". This resulted in a four-level categorical variable.
**Years since qualification**	Continuous variable. Derived from free-text responses to the question "In which year did you obtain your primary professional qualification?". The question was asked to anyone who reported working in one of the following professions: Arts therapist, Biomedical scientist, Chiropodist/Podiatrist, Clinical scientist, Dietician, Hearing aid dispenser, Occupational therapist, Operating department practitioner, Orthoptist, Physiotherapist, Practitioner psychologist, Prosthetist / Orthotist, Radiographer, Speech and language therapist, Other Allied Health Professional role, Paramedic, Clinical dental technician, Dental Hygienist, Dental nurse, Dental technician, Dentist, Other dental role, Doctor, Advanced Nurse Practitioner, Midwife, Nurse, Nursing Associate, Other nursing and midwifery role , Pharmacist, Pharmacy technician, Other pharmacy role, Dispensing optician, Optometrist, Other Optical role. Responses were used to calculate years since gaining a primary professional qualification by subtracting the reported year of qualification from 2021 (the year of questionnaire administration)<br><br>We coded as missing participants who reported a year of qualification later than their questionnaire completion date or whose qualification date was before 1950. As a data validation step, we calculated the difference between a participants reported age and the number of years they reported being qualified and coded as missing anyone who had indicated they gained their professional qualification before being 18 years old.
**Educational level**	Categorical variable. Derived from the question " What is the highest level of education you have completed?". Participants could choose from the following options: Primary (up to 11 years), Secondary (11 to 16 years), Post-secondary (16-18 years), Other qualifications below degree level (e.g. nursing diploma), Undergraduate degree or equivalent (e.g. BA, BSc, medical or nursing degree), Master's degree or equivalent (e.g. MSc, Ma), Doctorate level (e.g. PhD, MD). These were collapsed into three categories: Undergraduate degree or lower, masters, and doctorate.

Supplementary Table 2.
Derivation of variables from questionnaire data

Supplementary Table 3. **Contingency table of exposure and other covariates**

Variable	White (n=4751)	Asian (n= 586)	Black (n=186)	Mixed (n=192)	Other (n=56)	P value
**Sex**						
Male	693 (14.6)	129 (22.0)	39 (21.0)	26 (13.5)	14 (25.0)	0.000
Female	4058 (85.4)	457 (78.0)	147 (79.0)	166 (86.5)	42 (75.0)	
**AfC Band**						
Band 5 or below	922 (19.4)	133 (22.7)	51 (27.4)	46 (24.0)	14 (25.0)	0.002
Band 6	1810 (38.1)	187 (31.9)	61 (32.8)	77 (40.1)	19 (34.0)	
Band 7	1318 (27.7)	149 (25.4)	49 (26.3)	46 (24.0)	13 (23.2)	
Band 8 and above	701 (14.8)	117 (20.0)	25 (13.4)	23 (12.0)	10 (17.9)	
**Years qualified**						
Median, (IQR)	17 (8, 29)	15 (6, 24)	15 (7, 25)	12 (5, 20)	15 (6, 28)	0.0001
**Born Abroad**						
UK Born	4258 (89.6)	242 (41.3)	67 (36.0)	147 (76.6)	8 (14.3)	0.000
Oversea Born	493 (10.4)	344 (58.7)	119 (64.0)	45 (23.4)	48 (85.7)	
**Education level**						
Undergrad or lower	3291 (69.3)	332 (56.7)	116 (62.4)	133 (69.3)	34 (60.7)	0.000
Masters	1252 (26.4)	205 (35.0)	61 (32.8)	47 (24.5)	17 (30.4)	
Doctorate	208 (4.4)	49 (8.4)	9 (4.8)	12 (6.3)	5 (8.9)	
**Job**						
Nurses/NA/Midwives	1809 (38.1)	148 (25.3)	70 (37.6)	47 (24.5)	21 (37.5)	0.000
AHPs	2139 (45.0)	292 (49.8)	79 (42.5)	111 (57.8)	27 (48.2)	
Pharmacy	60 (1.3)	56 (9.6)	7 (3.8)	4 (2.1)	1 (1.8)	
Healthcare scientist	369 (7.8)	67 (11.4)	25 (13.4)	15 (7.8)	4 (7.1)	
Ambulance	348 (7.3)	13 (2.2)	4 (2.2)	15 (7.8)	2 (3.6)	
Optical	26 (0.5)	10 (1.7)	1 (0.5)	0	1 (1.8)	

Supplementary Table 3.
Contingency table of exposure and other covariates cross-tabulated with other outcome measure and compared distributions using Kruskal-Wallis tests for continuous variables and chi-squared tests for categorical variables. The figures are described in absolute numbers and percentage unless otherwise stated. IQR – interquartile range, NA – Nursing Associate, AHPs – Advanced Healthcare Practitioners

Supplementary Table 4. Detailed description of variables

	Cut point 1<br>Band 5 vs Band 6 and above		Cut point 2<br>Band 6 and below vs Band 7 and above		Cut point 3<br>Band 7 and below vs Band 8 and above	
**Ethnicity/migrant status group**						
**White overseas**	0.8 (0.7, 1.0)	<0.024	0.8 (0.7, 1.0)	<0.024	0.8 (0.7, 1.0)	<0.024
**Asian UK**	0.8 (0.7, 1.0)	<0.270	1.3 (0.9, 1.8)	<0.092	1.2 (0.8, 1.8)	<0.381
**Asian overseas**	0.6 (0.5, 0.75)	<0.000	0.6 (0.5, 0.8)	<0.000	0.6 (0.5, 0.8)	<0.000
**Black UK**	1.2 (0.8, 1.9)	<0.449	1.2 (0.8, 1.9)	<0.449	1.2 (0.8, 1.9)	<0.449
**Black overseas**	0.5 (0.4, 0.7)	<0.000	0.5 (0.4, 0.7)	<0.000	0.52 (0.4, 0.7)	<0.000
**Mixed UK**	0.9 (0.7, 1.2)	<0.438	0.9 (0.7, 1.2)	<0.438	0.9 (0.7, 1.2)	<0.438
**Mixed overseas**	0.7 (0.4, 1.3)	<0.251	0.72 (0.4, 1.3)	<0.251	0.7 (0.4, 1.3)	<0.251
**Job role**						
**AHPs**	2.8 (2.4, 3.2)	<0.000	1.8 (1.6, 2.1)	<0.000	1.3 (1.1, 1.6)	<0.000
**Pharmacy**	2.9 (1.6, 5.1)	<0.000	5.3 (3.3, 8.4)	<0.000	7.9 (5.1, 2.1)	<0.000
**Healthcare Scientist**	3.1 (2.0, 4.3)	<0.000	1.6 (1.3, 2.1)	<0.000	1.4 (1.0, 1.8)	<0.048
**Ambulance**	3.7 (2.8, 5.0)	<0.000	0.5 (0.4, 0.8)	<0.001	0.8 (0.4, 1.5)	<0.487
**Optical**	4.0 (2.1, 7.7)	<0.000	4.0 (2.1, 7.7)	<0.000	4.0 (2.1, 7.7)	<0.000
**Education level**						
**Masters**	3.1 (2.6, 3.8)	<0.000	4.81 (4.2, 5.5)	<0.000	5.2 (4.4, 6.2)	<0.001
**Doctorate**	49.7 (36.5, 67 .5)	<0.000	49.7 (36.5, 67 .5)	<0.000	49.7 (36.5, 67 .5)	<0.001
**Country of training**						
**Born abroad & trained UK**	0.8 (0.6, 0.9)	<0.001	0.8 (0.6, 0.9)	<0.001	0.8 (0.6, 0.9)	<0.001
**Born & trained abroad**	0.8 (0.6, 0.9)	<0.001	0.5 (0.4, 0.7)	<0.001	0.8 (0.6, 0.9)	<0.001

Supplementary Table 4.
Adjusted odds ratios, 95% Confidence Intervals and p-value across different AfC cut points for ethnicity, migration status, years qualified, job roles and education level. NA – Nursing Assistants, AHP – Advanced Healthcare Practitioner.
